# A Gene That Directs the Regeneration of Injured Muscle from Adult Stem Cells

**DOI:** 10.1371/journal.pbio.0020135

**Published:** 2004-05-11

If the United States' Human Cloning Prohibition Act of 2003 (H.R. 534) becomes law, American researchers practicing any form of cloning could face up to ten years in prison and a minimum $1 million fine. The bill criminalizes a research procedure, called somatic cell nuclear transfer, that involves removing the DNA from a fertilized egg and replacing it with the DNA of a body (soma) cell. While the procedure could theoretically be used to clone a human being, used therapeutically its great promise lies in yielding a renewable source of stem cells to repair and regenerate tissue damaged by disease or injury. Embryonic stem cells appear most suited to this task, but some researchers are finding that adult stem cells could perform similar duties in certain tissues. And adult stem cells, it appears, are responsive to genetic manipulation. H.R. 534 does not threaten researchers working with adult stem cells.[Fig pbio-0020135-g001]

**Figure pbio-0020135-g001:**
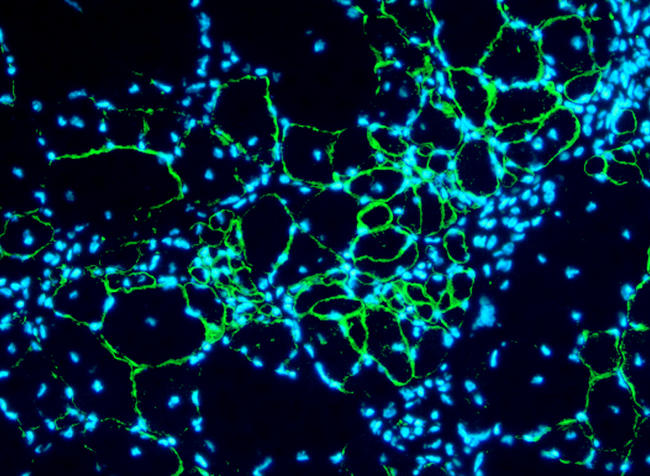
Pax7-infected stem cells rescue dystrophin expression

The precise origin of adult stem cells is unclear, though some propose that they are “set aside” during embryonic development and sequestered in mature tissue. These cells, which can make identical copies of themselves or give rise to specialized cells, serve primarily to replace damaged or injured cells. Skeletal muscle has a remarkable capacity to regenerate following exercise or injury and harbors two different types of adult stem cells to accomplish the job: satellite cells and adult stem cells that can be isolated as side population (SP) cells. Like embryonic stem cells, the adult cells commit to a certain fate once particular genes are activated.

It was thought that only satellite cells could mediate skeletal muscle regeneration until recently, when scientists found that adult stem cells not only participate in muscle tissue regeneration but also spawn satellite cells. A certain population of these stem cells, which are recognized by the cell surface proteins CD45 and Sca1 (stem cell antigen-1), is involved in normal muscle tissue repair, but is only triggered into the muscle cell development pathway by injury. The question then arises: what molecular factors turn these adult stem cells into muscle cells? Now Michael Rudnicki and colleagues have shown that one gene, *Pax7,* plays a crucial role in directing the differentiation of these adult stem cells into skeletal muscle cells.

In previous studies, Rudnicki's group demonstrated that *Pax7* is required to turn adult stem cells into myogenic cells during regeneration. Here, the researchers worked with mouse models and in vitro experiments to investigate which cell populations *Pax7* targets and how the gene initiates muscle cell formation in injured tissue. They show that CD45:Sca1 cells taken from regenerating muscle in mice lacking the *Pax7* gene could not become muscle cells. And they show that by putting *Pax7* back into CD45:Sca1 cells taken from uninjured muscle, they can generate a population of proliferating myoblasts that readily differentiate into muscle cells. When CD45:Sca1 cells engineered to express *Pax7* proteins were injected into the muscles of mice lacking dystrophin (the protein defective in muscular dystrophy), the cells differentiated, forming dystrophin-expressing muscle cells in the defective muscle. This shows that engineered “donor cells” can differentiate in living tissue and help repair dystrophic muscle. When the researchers injected *Pax7* (using a gene therapy virus) into the damaged muscle of mice lacking *Pax7*, they observed the production of muscle-forming cells that not only gave rise to differentiated muscle cells, but also aided in tissue repair.

The researchers argue that these results “unequivocally establish” *Pax7* as a key regulator of muscle cell differentiation in specific populations of adult stem cells during muscle tissue regeneration. If therapeutic strategies that activate *Pax7* in adult stem cells can turn them into muscle cells, effectively replenishing injured or diseased muscle tissue, there's hope of reversing the debilitating effects of progressive muscle-wasting diseases. Though the clinical efficacy of such an approach will require intensive investigation, the results on these adult stem cells are encouraging—especially in this political climate.

